# Anomalous Left Main Coronary Artery From the Right Sinus of Valsalva

**DOI:** 10.7759/cureus.35709

**Published:** 2023-03-02

**Authors:** Hamza Chraibi, Amina Samih, Nouhaila Lahmouch, Jamila Zarzur, Mohamed Cherti

**Affiliations:** 1 Cardiology B Department, Ibn Sina University Hospital, Mohammed V University, Rabat, MAR

**Keywords:** coronary artery disease, coronary angiography, acute coronary syndrome, coronary circulation, coronary artery anomalies

## Abstract

Anomalous coronary arteries are rare, mostly benign anatomic abnormalities. Anomalous origin of the left main coronary artery from the right sinus of Valsalva (LCA-RSV) is a rare variant that may lead to myocardial ischemia or sudden cardiac death. We present the case of a 49-year-old patient with a history of type 2 diabetes and smoking who presented to the emergency department with acute chest pain and was diagnosed with inferior ST-elevation myocardial infarction (STEMI). A transthoracic echocardiogram demonstrated inferolateral wall motion abnormalities of the left ventricle. The patient underwent cardiac catheterization that showed an anomalous left main coronary artery originating from the right sinus of Valsalva, alongside atherosclerotic triple-vessel disease. He was discharged home on medical management, including dual antiplatelet therapy, beta blockers, and statins, with scheduled follow-up.

## Introduction

Coronary artery anomalies are incidentally found in 0.2% to 1.3% of patients undergoing coronary angiography and 0.3% of autopsies [1]. While mostly benign and asymptomatic, they lead in rare cases to life-threatening conditions such as myocardial ischemia, arrhythmias, syncope, and sudden death.

The prevalence of these anomalies varies widely. Anomalous origin of the left main coronary artery from the right sinus of Valsalva (LCA-RSV) is reported in 0.003% to 0.017% of cases and represents one of the rarest variations of coronary circulation [1].

We report the case of a young male patient who was diagnosed with acute coronary syndrome and whose coronary angiography showed LCA-RSV. We discuss the different diagnostic and management options.

## Case presentation

A 49-year-old male with a history of type 2 diabetes and smoking presented to the emergency department. He complained of transient chest pain beginning two weeks before. He sought medical attention because the current episode was longer-lasting and more intense than the ones before. Initial physical examination showed that his heart rate was 74 beats per minute and his blood pressure 113/79 mmHg. His respiratory rate was 18 breaths per minute at rest, and O2 saturation at 99%. Auscultation was normal.

Electrocardiogram at admission showed sinus tachycardia with signs of ischemia in inferior leads, ST-segment elevation, Q waves, and inversed T waves, confirming the diagnosis of ST-elevation myocardial infarction (STEMI). Chest radiograph found an enlargement of the cardiac silhouette. Biological findings included high levels of ultrasensitive troponin (3210 ng/L). Blood count and electrolytes were normal. Initial echocardiographic evaluation demonstrated inferolateral wall motion abnormalities of the left ventricle, with a mildly reduced ejection fraction (46%).

**Figure 1:**
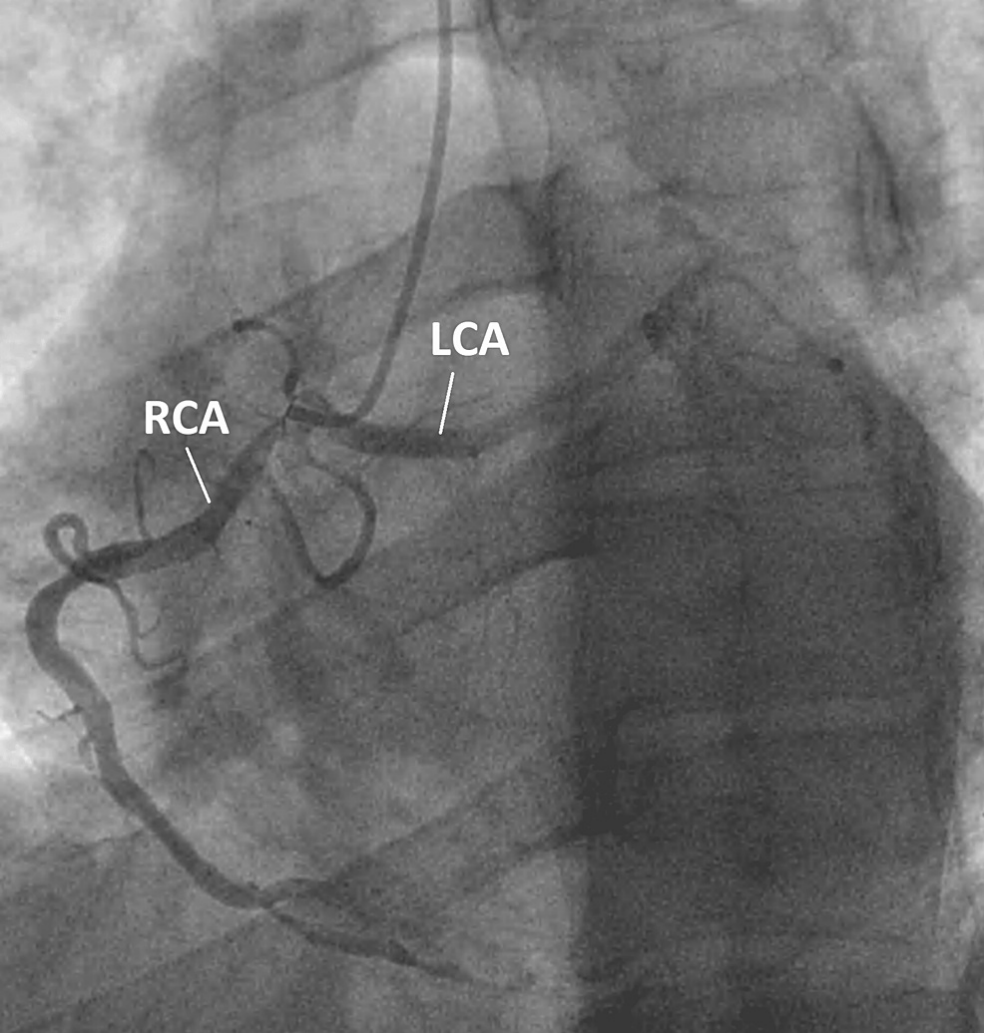
Coronary angiography (left oblique anterior view) Anomalous origin of the left coronary artery from the right sinus of Valsalva. LCA: left main coronary artery; RCA: right coronary artery

The patient underwent coronary angiography three hours after admission as he presented late. It showed a left main coronary artery originating from the right sinus of Valsalva and trifurcating into three main vessels. We noted triple-vessel atherosclerotic disease; the circumflex artery (CX) was occluded on its distal portion, and significant lesions were also found on the right coronary artery (RCA), the left anterior descending artery (LAD), and the main diagonal (Figure 1). Medical therapy was initiated, consisting of dual antiplatelet therapy, beta blockers and statins. After an uncomplicated hospital course, the patient was discharged home. He was lost to follow-up and no further exploration could be performed.

## Discussion

LCA-RSV is a very rare anomaly, found in 0.003% to 0.017% of all angiographies performed [1]. Its classification is based on the course taken by the LCA; four entities exist: 1) the septal course beneath the right ventricular infundibulum, 2) the anterior course in the left ventricular free wall, 3) the retro-aortic course, 4) the inter-arterial course between the aorta and pulmonary trunk [2]. Among all pathways, the inter-arterial course is the least common but most malignant, responsible for arrhythmias or sudden deaths. It was postulated that the LCA is squeezed between the major vessels when dilated during intense activities, causing acute myocardial ischemia.

Clinical presentation varies. Only 20% of patients present with symptoms such as angina, dyspnea, or syncope. Sudden cardiac death is also a common presentation, mostly in young athletes. Sometimes, the anomaly is incidentally found in patients with other causes of ischemia, mainly atherosclerosis. Electrocardiographic findings are nonspecific, such as ST-wave elevation or depression, T-wave anomalies, or arrhythmias such as ventricular tachycardia or fibrillation [3].

**Figure 2:**
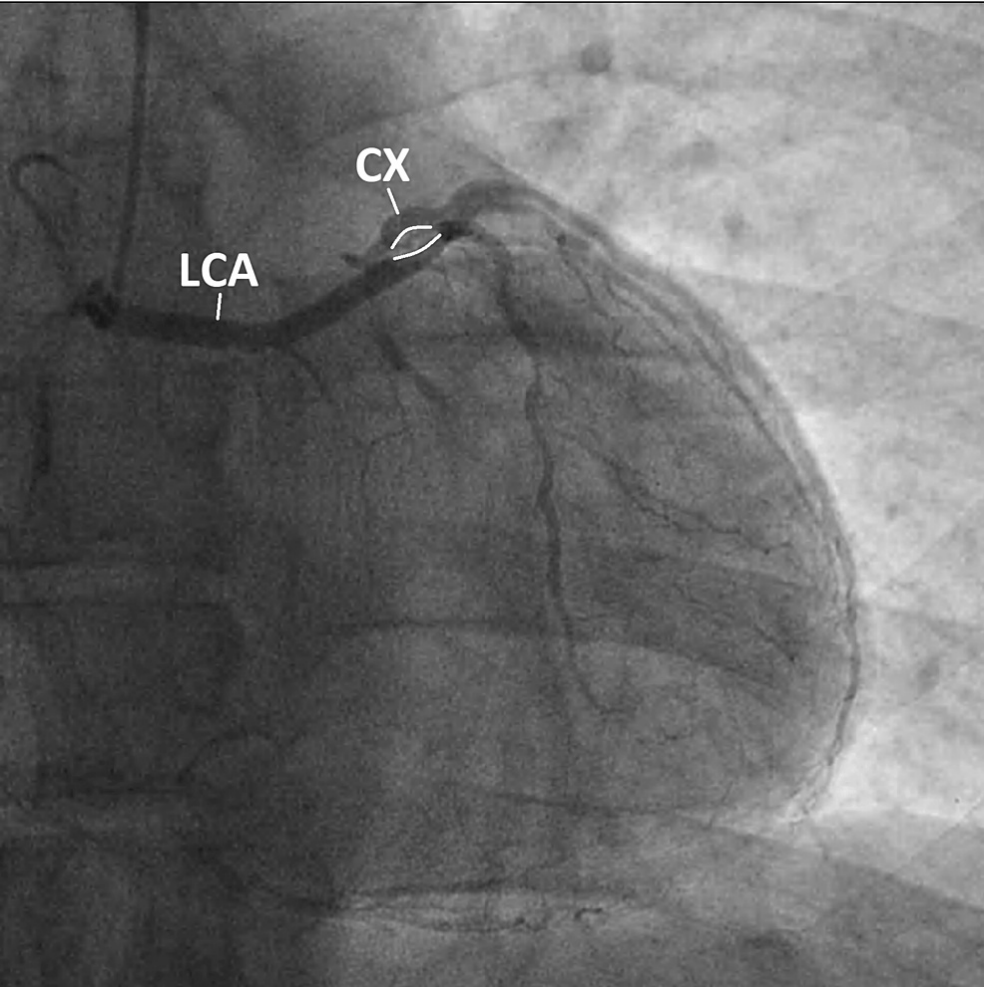
Coronary angiography (cranial right oblique anterior view) “Eye-like” pattern, drawn in white, formed by the circumflex artery (superior) and the left coronary artery (inferior), in favor of a septal course. LCA: left main coronary artery; CX: circumflex artery

**Figure 3:**
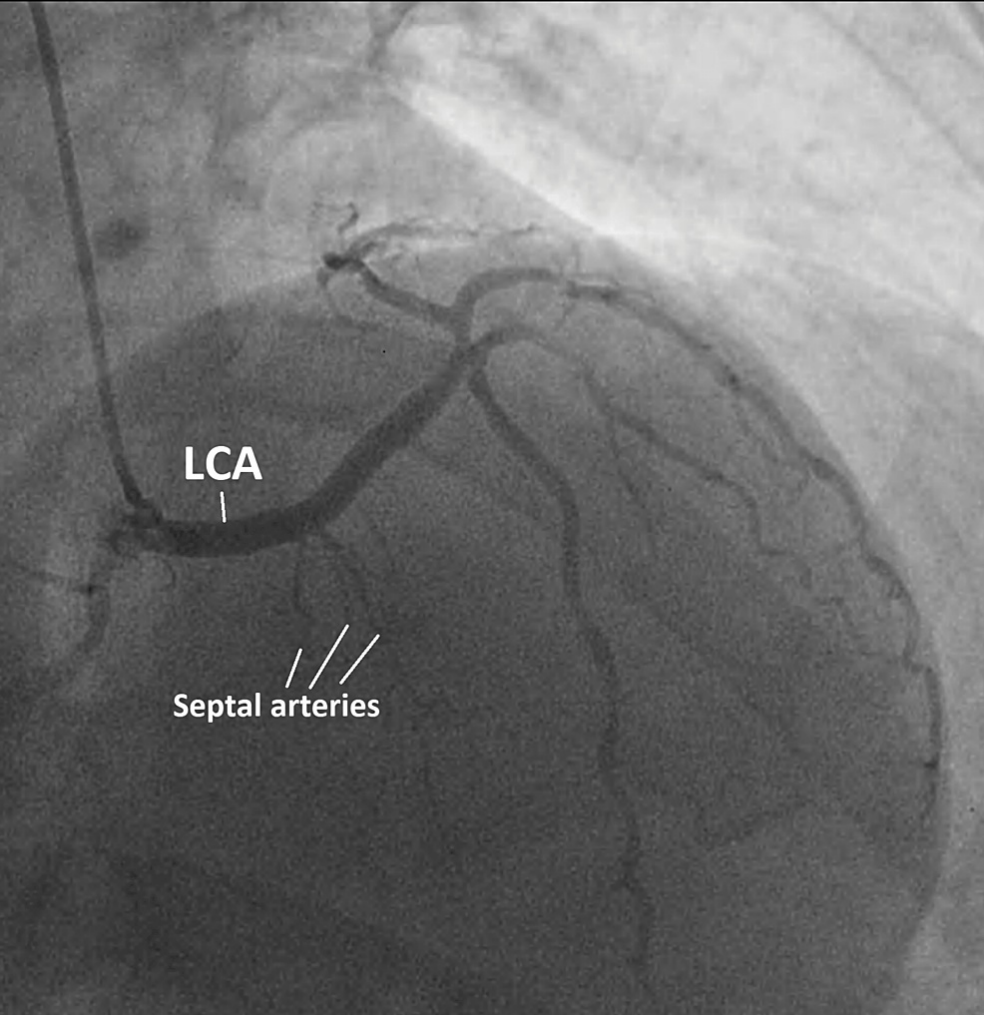
Coronary angiography (modified right oblique anterior view) Septal arteries originating from the left coronary artery, in favor of a septal course. LCA: left main coronary artery

During coronary angiography, it is possible to distinguish between the four courses. In the right anterior oblique (RAO) view, one can use the “dot and eye” method proposed by Serota et al. [2]. In the septal course, an ellipse or eye-like pattern, with the superior and inferior margins being formed by the CX and LCA, respectively, can be seen. Septal arteries originating from the LCA further validate this hypothesis. In our patient’s coronary angiography, an ellipse formed by the CX superiorly and LCA inferiorly is observed in the RAO view (Figure 2). In the cranial RAO view, septal perforators originating from the LCA confirm the septal variant (Figure 3).

The diagnosis of this anomaly raises several clinical questions. An etiological assessment must be considered in patients presenting the symptoms mentioned, especially athletes, including an electrocardiogram, Holter monitoring, and an echocardiography performed by an expert. If echocardiography identifies two normally localized coronary ostia, no further investigation is required. However, if it is inconclusive, other investigations, including magnetic resonance imaging and computed tomography imaging, are recommended. An ischemia test, coronary angiography to highlight the coronary lesion or even intracoronary imaging should be performed if the diagnosis is confirmed [4,5].

The 2018 AHA/ACC (American Heart Association/American College of Cardiology) guidelines recommend surgical repair in all patients with LCA-RSV (class I when ischemia is present and class IIa when it’s not) [6]. In our patient’s case, STEMI etiology was probably atherosclerosis and not the anomaly itself.

## Conclusions

Our patient is a unique case of LCA-RSV, with the entirety of the coronary circulation originating from a single aortic sinus. It is a rare, potentially deadly malformation. Invasive angiography is the diagnostic modality of choice. Current guidelines recommend surgical correction regardless of ischemia or symptoms, but management is tricky when associated with coronary artery disease.
